# A case series on the role of multimodality imaging in the diagnosis, risk stratification and complications of left ventricular pseudoaneurysm

**DOI:** 10.4102/sajr.v30i1.3530

**Published:** 2026-10-22

**Authors:** Nadeem Ur Rahman, S. Mac Win

**Affiliations:** 1 Department of Radiodiagnosis, Chettinad Academy of Research and Education, Chennai, India; 2 Department of Radiodiagnosis, Christian Medical College Vellore, Vellore, India

**Keywords:** left ventricular pseudoaneurysm, systemic embolism, left ventricular thrombus, computed tomography, acute myocardial infarction, cardiac magnetic resonance imaging, true aneurysm, multimodality imaging

## Abstract

Left ventricular (LV) pseudoaneurysm is an uncommon, life-threatening sequela of myocardial injury, defined by contained rupture of the ventricular free wall by pericardium or scar tissue rather than intact myocardium. Unlike true aneurysms, pseudoaneurysms carry a rupture risk of 30%–45% and a 1-week mortality of 48% in medically managed patients, making prompt diagnosis and accurate differentiation critical for patient survival. Multimodality imaging – chest radiography, echocardiography, CT and cardiac MRI (CMR) – is indispensable in assessing morphology, haemodynamic impact, tissue characterisation, thrombus burden and systemic complications. Cardiac MRI serves as the reference standard for differentiating pseudoaneurysm from true aneurysm and for risk stratification.

**Contribution:**

This case series highlights the pivotal role of imaging in the surgical and conservative management planning of LV pseudoaneurysm.

## Introduction

Left ventricular (LV) pseudoaneurysm is an uncommon but clinically significant sequela of myocardial injury, most often following transmural infarction or cardiac surgery. It is defined by rupture of the ventricular free wall contained by the pericardium or organised scar rather than viable myocardium, in contrast to a true aneurysm, which involves remodelling of intact, but thinned, myocardium.^1,2^ The incidence of LV pseudoaneurysm following myocardial infarction is reported at 0.2%–0.3% of all patients, though this figure likely reflects underdiagnosis given the often non-specific clinical presentation.^3^ Pseudoaneurysms carry a high risk of rupture and systemic thromboembolism, with reported rupture rates ranging from 30% to 45%, mandating early recognition and often, urgent surgical management.^1,4,5^

Imaging plays a central role in diagnosis and risk stratification. Echocardiography is frequently the first-line tool, though its sensitivity may be limited in distinguishing pseudoaneurysm from true aneurysm, particularly in patients with poor acoustic windows or posteriorly located lesions. Transoesophageal echocardiography (TEE) offers superior visualisation of posterior and basal segments in such cases, albeit at the cost of semi-invasiveness.^5^ Contrast-enhanced CT (CECT) offers excellent spatial resolution, clearly depicting aneurysmal morphology, the narrow neck, the orifice-to-cavity ratio (characteristically less than 0.5 in pseudoaneurysm) and associated mural thrombus or systemic embolic sequelae.^4,6^ Cardiac MRI (CMR) provides additional advantages by enabling tissue characterisation, with late gadolinium enhancement (LGE) sequences demonstrating discontinuity of the myocardial wall with pericardial containment – findings diagnostic of pseudoaneurysm. Significant delayed pericardial enhancement on CMR is a characteristic and discriminating feature of LV pseudoaneurysm, demonstrating 100% sensitivity and 83% specificity in an initial series.^7^ Additionally, the ‘myocardial cut-off sign’ – defined as a greater than 50% reduction in aneurysm sac wall thickness within 1 cm of the neck – is 91% sensitive and 97% specific for pseudoaneurysm on both CT and CMR.^8^ Cardiac MRI also provides information on myocardial viability and scarring, which guides risk stratification and surgical planning.^7,9,10^

A multimodality imaging approach is therefore essential in LV pseudoaneurysm, not only to establish the diagnosis, but also to differentiate it from true aneurysm, evaluate associated complications and direct timely therapy. This case series illustrates across three patients with contrasting clinical profiles, how each imaging modality contributes uniquely to the diagnostic and management pathways.

## Ethical considerations

All procedures performed in this case series involving human participants were in accordance with the ethical standards of the institutional and/or national research committee and with the 1964 Helsinki Declaration and its later amendments or comparable ethical standards. Written informed consent was obtained from all individual participants involved in the study.

## Case presentations

### Case 1

A 45-year-old male presented with progressive exertional dyspnoea, orthopnoea and pedal oedema over several weeks. There was a past history of multiple transient ischaemic attacks and recurrent abdominal pain. He had multiple comorbidities, including hypertension and diabetes mellitus, and was poorly compliant with oral medications. On examination, he was tachycardic with an elevated jugular venous pressure, a displaced apical impulse and a systolic murmur at the apex.

Chest radiography (Figure 1a) demonstrated cardiomegaly with a focal bulge along the left cardiac border, suggesting an abnormal ventricular contour. Contrast-enhanced CT of the chest (Figure 1b) revealed a large LV pseudoaneurysm arising from the mid and basal anterolateral wall, measuring approximately 6 cm × 7 cm, with a narrow neck and eccentric mural thrombus. Abdominal CECT (Figure 2a) demonstrated wedge-shaped hypodense areas in the kidney, consistent with embolic infarction and correlating with the patient’s episodes of abdominal pain. Non-contrast CT (NCCT) of the brain (Figure 2b–d) revealed chronic infarcts, confirming systemic embolisation as a complication of the pseudoaneurysm.

**FIGURE 1:**
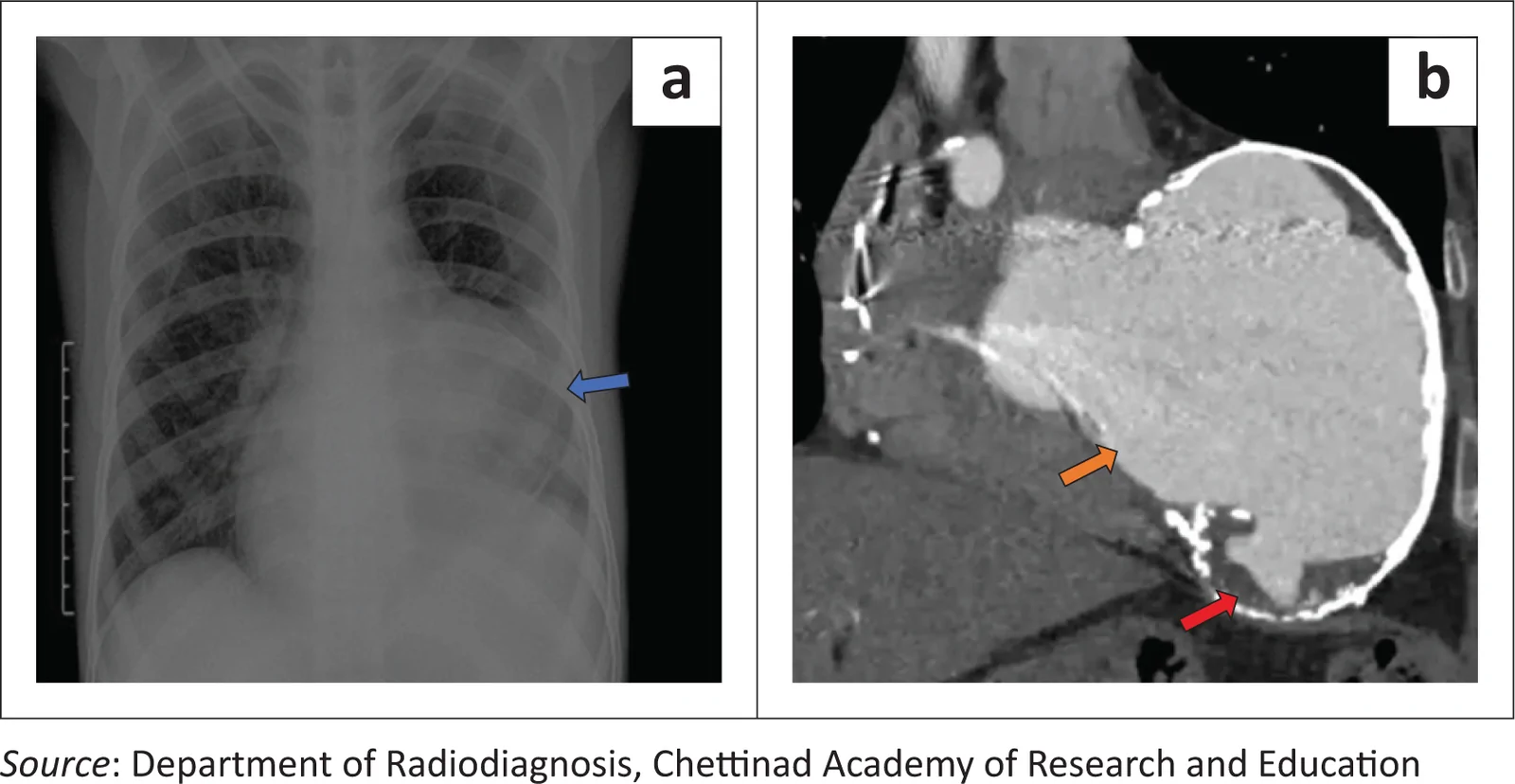
(a) Chest radiograph demonstrating cardiomegaly with a localised bulge in the left cardiac border and faint calcification (blue arrow). (b) Contrast-enhanced CT thorax reveals a large aneurysmal outpouching arising from the basal lateral wall (orange arrow) with intraluminal eccentric thrombus (red arrow) layering along the periphery of the aneurysmal outpouching and rim calcification.

**FIGURE 2:**
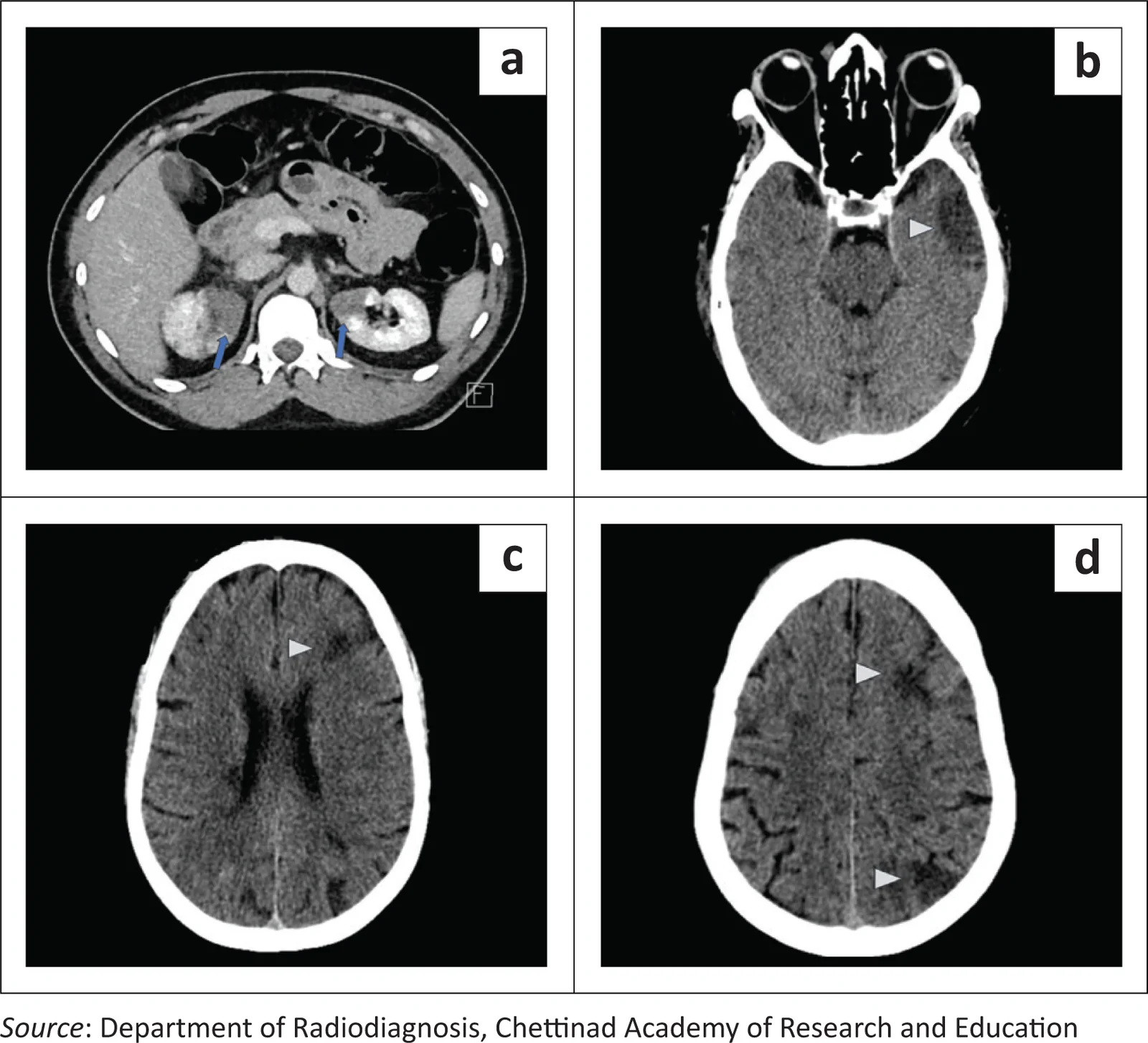
(a) Contrast-enhanced CT abdomen depicts small, peripheral, wedge-shaped areas of hypo-enhancement (blue arrows) in the upper poles of both kidneys, consistent with embolic renal infarcts. (b, c, d) Non-contrast CT brain revealing multifocal, hypodense areas (white arrowheads) in the left cerebral hemisphere without mass effect or vasogenic oedema, consistent with chronic cerebral infarctions.

Based on multimodality imaging findings, a diagnosis of LV pseudoaneurysm with mural thrombus and systemic embolic complications was established. The patient was initiated on systemic anticoagulation with heparin, subsequently bridged to oral warfarin, to reduce the risk of further embolic events from the large intrasaccular thrombus. After hemodynamic stabilisation, he underwent surgical repair under cardiothoracic guidance. The pseudoaneurysmal sac at the mid and basal anterolateral wall was resected, and the defect was closed with a Dacron patch. Postoperatively, he was maintained on anticoagulation and standard heart failure therapy, with a satisfactory recovery.

### Case 2

A 55-year-old male with a history of prior myocardial infarction presented with recurrent palpitations and episodic chest pain over the preceding 3 months. He was on standard medical therapy for ischaemic heart disease. Clinical examination was unremarkable except for an irregular pulse; electrocardiography demonstrated *Q* waves in the inferior leads with intermittent ventricular ectopic beats.

Transthoracic echocardiography (TTE) revealed a saccular outpouching arising from the basal inferior segment of the left ventricle, with a narrow neck (Figure 3). Doppler imaging indicated turbulent flow (image not available). Left ventricular systolic function was moderately reduced, and a presumptive diagnosis of pseudoaneurysm was made; however, definitive differentiation of pseudoaneurysm from true aneurysm could not be established on echocardiography alone.

**FIGURE 3:**
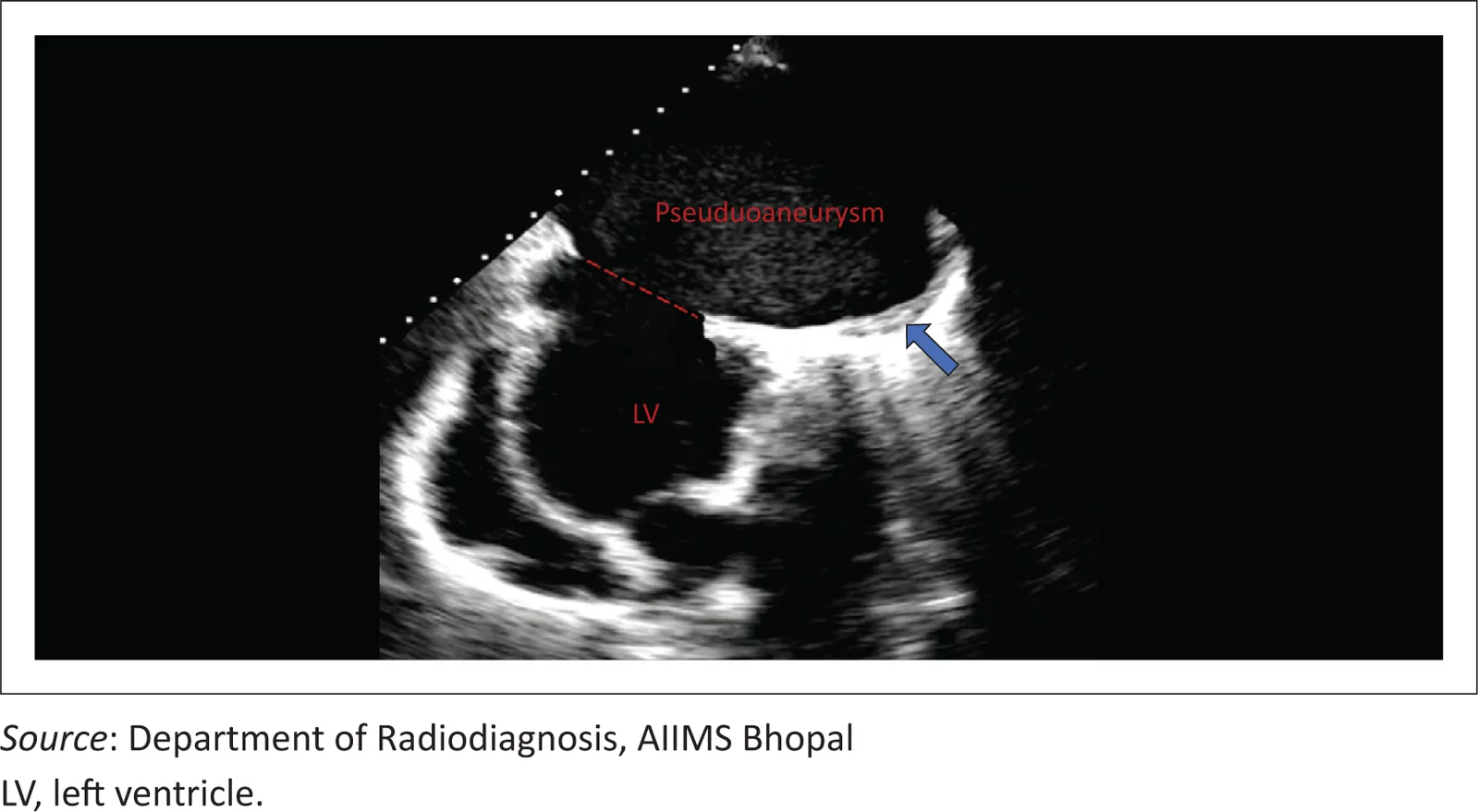
Transthoracic echocardiography showing a large pseudoaneurysmal sac with a narrow neck (red dashed line) and echogenic thrombus (blue arrow) layering along the dependent portion of the aneurysmal cavity.

Cardiac MRI was subsequently performed for definitive characterisation and to estimate clot burden. It confirmed a 5 cm × 6.5 cm aneurysmal outpouching from the basal and mid-inferior wall with a narrow neck. Cine steady-state free precession (SSFP) sequences in short-axis, four-chamber and oblique axial views demonstrated a large defect at the basal inferior myocardial segment extending into the mid-ventricular inferior wall, with a resultant aneurysmal outpouching contained by the pericardium. The cine sequences clearly demonstrated paradoxical systolic motion of the sac, consistent with pseudoaneurysm. Late gadolinium enhancement sequences demonstrated discontinuity of the myocardial wall with pericardial containment. Transmural scarring consistent with prior infarction was also identified (Figure 4a, Figure 4b and Figure 5).

**FIGURE 4:**
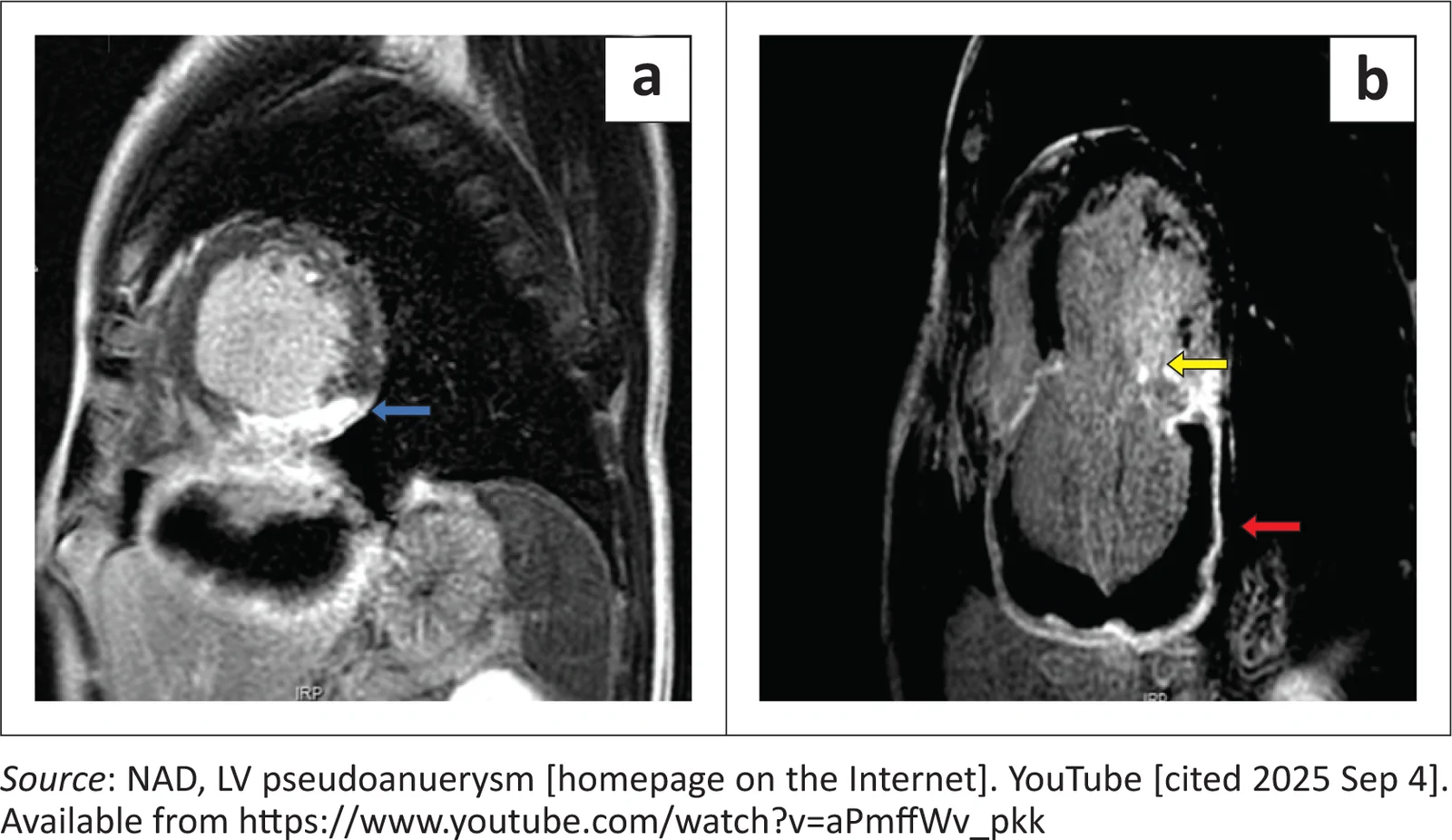
Late gadolinium enhancement imaging inversion recovery gradient-echo sequence (a and b), demonstrating discontinuity of the myocardial wall with pericardial containment and enhancement, consistent with left ventricular pseudoaneurysm (blue arrow – transmural enhancement at the basal inferior myocardium, red arrow – marked pericardial enhancement, yellow arrow – myocardial rupture at the basal inferior segment).

**FIGURE 5:**
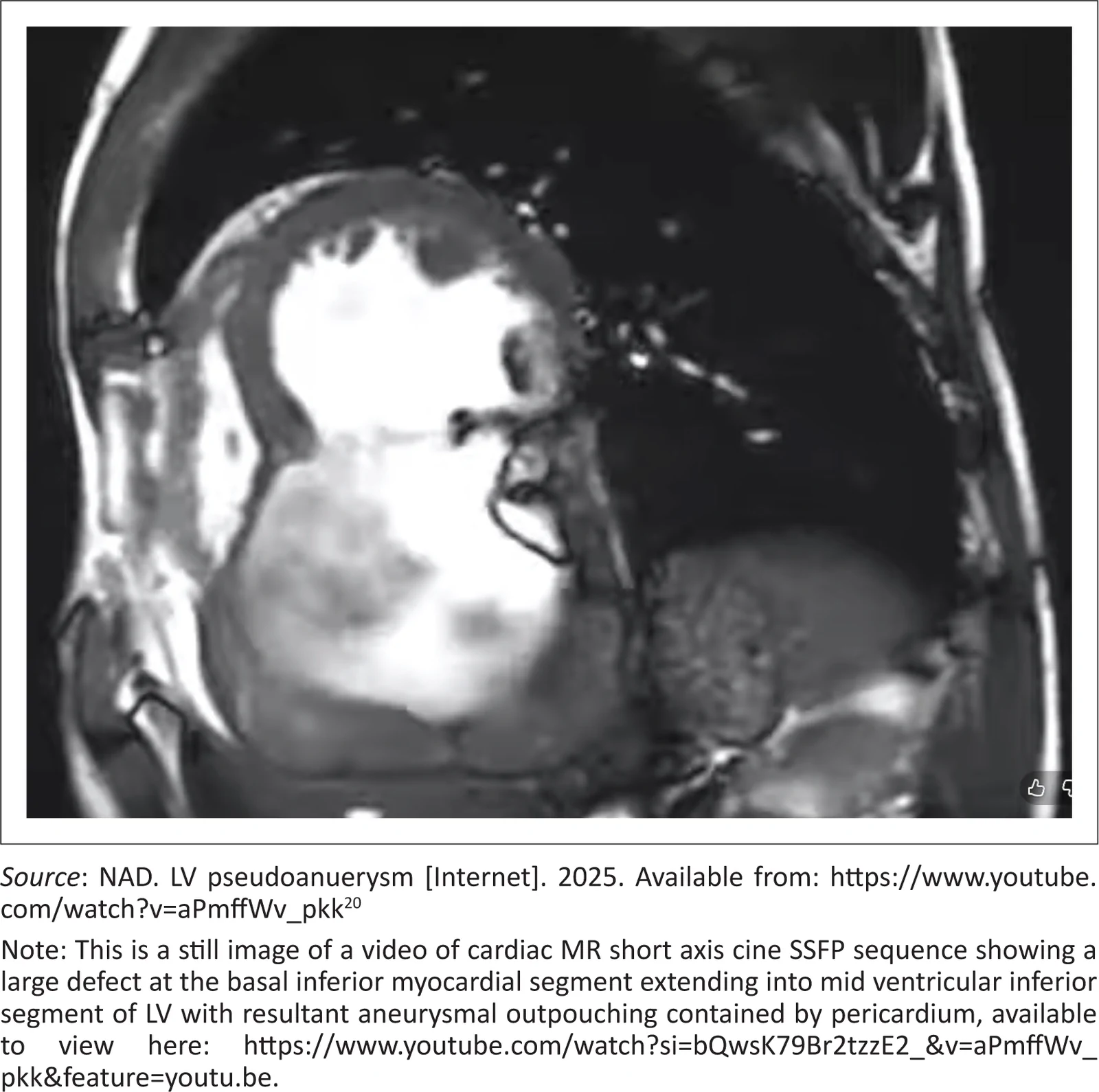
Cardiac MR- Short axis cine SSFP sequence shows large defect at the basal inferior myocardial segment extending into mid ventricular inferior segment of LV with resultant aneurysmal outpouching contained by pericardium.

The patient was referred for surgical management and underwent surgical repair under cardiopulmonary bypass; the pseudoaneurysmal sac at the basal and mid-inferior wall was resected, and the defect was closed with patch reconstruction. Postoperatively, he was maintained on anticoagulation and standard heart failure therapy, with an uneventful recovery.

### Case 3

A 76-year-old male with multiple comorbidities, including long-standing hypertension, diabetes mellitus and chronic kidney disease, presented with acute worsening of dyspnoea and chest discomfort. On admission, he was haemodynamically unstable, requiring inotropic support. Clinical examination revealed signs of acute heart failure, with bilateral basal crepitations and elevated jugular venous pressure.

Transthoracic echocardiography, performed in 2D mode, demonstrated a large pseudoaneurysmal cavity arising from the LV free wall, with a narrow neck communication into the ventricular cavity. Colour Doppler imaging confirmed turbulent flow across the neck (Figure 6a and Figure 6b). No additional advanced imaging could be performed due to the patient’s critical condition and haemodynamic instability.

**FIGURE 6:**
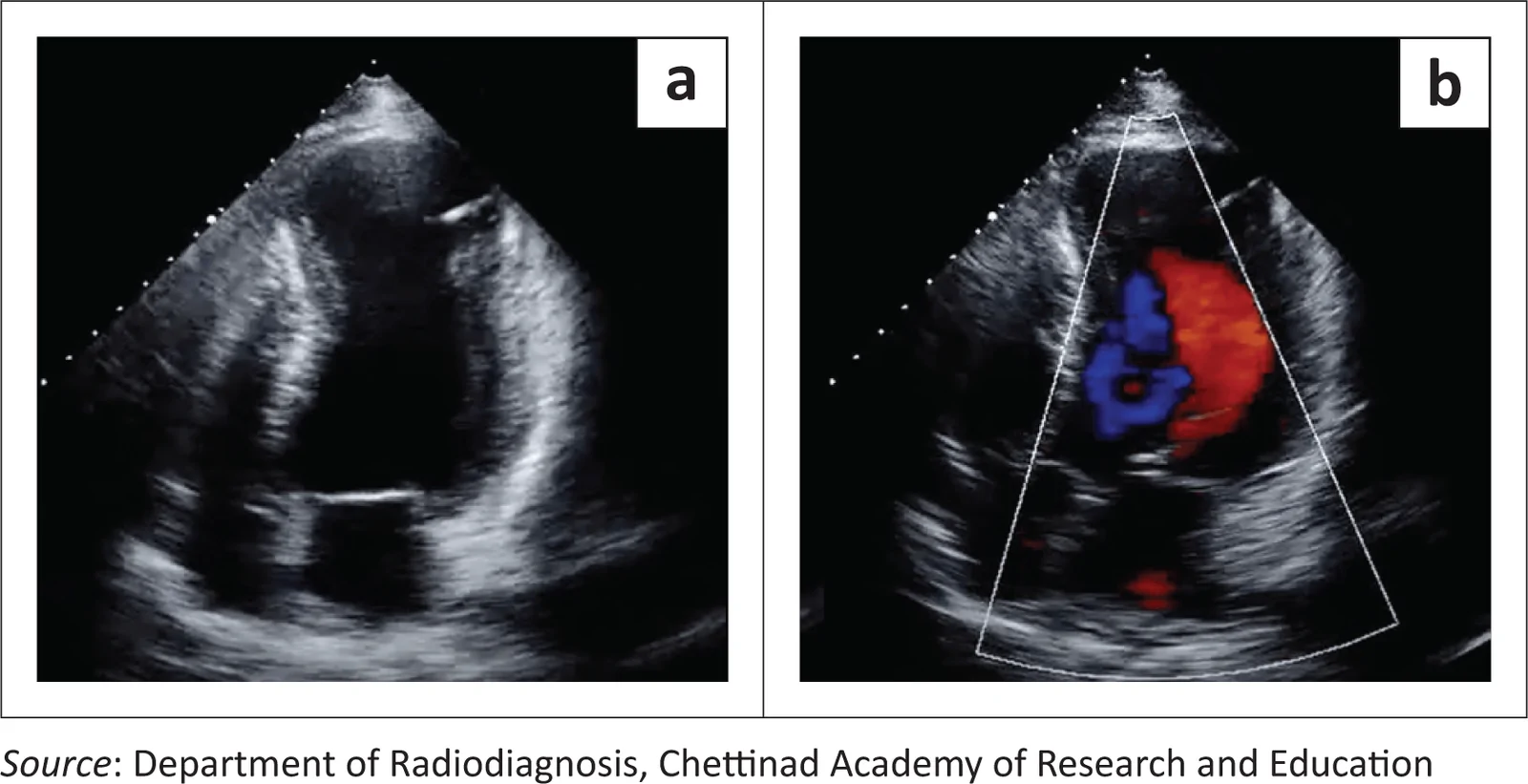
Transthoracic echocardiography (a) 2D image and (b) colour doppler image, demonstarting a large pseudoaneurysmal cavity with a narrow neck and turbulent colour doppler flow, consistent with left ventricular pseudoanuerysm.

Given the size of the pseudoaneurysm, the high risk of rupture and the patient’s prohibitive surgical risk, conservative supportive management was initiated. Despite maximal supportive measures, the patient’s condition deteriorated rapidly, and he succumbed to cardiovascular collapse within 24 hours of admission.

## Discussion

Left ventricular pseudoaneurysm is an uncommon but clinically important entity. The largest published series of 290 patients established that most LV pseudoaneurysms arise in the setting of myocardial infarction – particularly inferior wall infarction – and cardiac surgery. Congestive heart failure, chest pain and dyspnoea were the most frequently reported symptoms, while more than 10% of patients were asymptomatic.^1^ It represents a contained rupture of the ventricular wall where the breach is sealed by pericardium or organised thrombus rather than by viable myocardium.^1,2^ This structural vulnerability explains its propensity for rupture, making prompt recognition and accurate differentiation from a true aneurysm vital for guiding therapy. While true aneurysms are generally more stable and often managed conservatively, pseudoaneurysms carry a much higher risk of rupture and mandate early surgical repair.^1,5^

No single imaging technique can capture the full spectrum of information required for diagnosis and risk stratification in LV pseudoaneurysm. Instead, a multimodal approach is essential. Transthoracic echocardiography is usually the first investigation. It provides a rapid bedside assessment and can demonstrate key features such as a narrow neck (neck-to-sac ratio < 0.5), turbulent systolic flow across the defect and paradoxical outward motion of the sac.^5,11^ However, poor acoustic windows or suboptimal visualisation of posterior structures can limit its diagnostic accuracy. Transoesophageal echocardiography is often more sensitive in such situations, offering detailed images of the posterior and basal segments, though it is semi-invasive and less readily available.^5^

Cross-sectional imaging adds another dimension. Cardiac CT angiography (CCTA) offers high-resolution, three-dimensional visualisation, enabling precise measurement of the aneurysm and its orifice, as well as excellent delineation of relationships with adjacent coronary arteries or prosthetic material.^12^ The orifice-to-cavity ratio on CT, as noted above, remains a valuable morphological discriminator from a true aneurysm.^6^ ECG-gated multidetector CT plays a complementary role in distinguishing an LV pseudoaneurysm from an LV diverticulum, thereby expanding CT’s differential diagnostic utility in this setting.^6^ CT is particularly valuable in the acute setting or when rapid evaluation is needed, though its inability to characterise tissue limits its role compared with CMR.

Cardiac MRI has emerged as the reference standard for detailed structural and tissue assessment. Cine SSFP sequences demonstrate dynamic ventricular morphology and paradoxical sac expansion. At the same time, LGE provides critical tissue characterisation, including identification of intracavitary thrombus and quantification of myocardial scar burden, both of which directly inform surgical planning. Demonstrating myocardial discontinuity, pericardial enhancement and transmural infarction is diagnostic of pseudoaneurysm.^7,9^ The previously described myocardial cut-off sign likewise functions as a validated CT and CMR sign for distinguishing pseudoaneurysm from true aneurysm.^8^ Importantly, CMR is not only diagnostic, but also risk-stratifying. It defines infarct size, myocardial viability, thrombus burden and pericardial inflammation guiding surgical planning and long-term patient management.^7,9,10^

The clinical consequences of LV pseudoaneurysm are profound. Sudden rupture with cardiac tamponade - with a risk reported between 30% and 45% if untreated - is the most catastrophic complication and underlies the urgency of diagnosis.^1,3,13^ Beyond rupture, thrombus formation within the sac is common, and embolisation may lead to ischaemic stroke, visceral infarction or peripheral arterial occlusion.^14,15^ Arrhythmias, particularly ventricular tachyarrhythmias, are also well-recognised due to scar-related re-entry, contributing to sudden cardiac death in some patients.^16^ Over time, progressive cavity enlargement may lead to congestive heart failure, often compounded by distortion of the mitral apparatus and functional mitral regurgitation.^16,17^

Imaging is central not only to identifying these complications, but also to anticipating their clinical significance. Echocardiography can rapidly demonstrate haemodynamic compromise, while CT and CMR can identify thrombus, quantify sac size and evaluate its relationship to coronary arteries and valves. Cardiac MRI with LGE provides insights into the burden of scar and peri-aneurysmal tissue, information that can influence surgical decision-making and predict postoperative outcomes.^7,9,10^

Risk stratification of LV pseudoaneurysms hinges on two key questions: the risk of rupture and the lesion’s functional implications. Multimodality imaging addresses both. CT and CMR quantify the size and morphology of the sac. At the same time, CMR uniquely characterises the myocardial substrate, highlighting viable myocardium that may benefit from revascularisation and identifying scar that predisposes to arrhythmia. Cardiac MRI is particularly useful in surgical planning by enabling assessment of thrombus formation, the relationship of the aneurysm to adjacent cardiac structures and myocardial viability using gadolinium-enhanced sequences.^9^ These parameters help determine surgical urgency and shape long-term management, including anticoagulation, rhythm monitoring, and, in select cases, device therapy. Small, chronic pseudoaneurysms in high-risk surgical candidates may sometimes be managed conservatively, whereas large or symptomatic lesions almost invariably require repair.^14,18^

Management of LV pseudoaneurysm is challenging. Given the high risk of rupture, surgical repair with primary closure or patch reconstruction remains the preferred treatment in most patients.^14,18^ Conservative management may be considered in select situations – such as small (< 3 cm), asymptomatic pseudoaneurysms or patients deemed poor surgical candidates.^14,18,19,20^ However, conservative therapy requires close surveillance owing to the ongoing risk of rupture and embolism. Ultimately, management decisions must be individualised, balancing surgical risk with the lesion’s natural history.

### Conclusion

Left ventricular pseudoaneurysm remains a diagnostic and therapeutic challenge. A multimodal imaging strategy is indispensable: echocardiography provides an accessible first-line assessment; CT offers precise anatomic definition and complication detection; and CMR supplies unmatched tissue characterisation and risk stratification information. The three presented cases demonstrate that the imaging contribution varies by clinical context – CT is particularly powerful in detecting systemic embolic complications. At the same time, CMR is indispensable for differentiating it from a true aneurysm, and even when advanced imaging is not feasible, echocardiography provides sufficient diagnostic information to guide management decisions, including the difficult conclusion that surgical repair is not viable. Recognising complications such as rupture, thromboembolism, arrhythmias and progressive heart failure is vital. Integrating imaging modalities ensures timely diagnosis, guides the choice between surgical or conservative therapy and improves patient outcomes in this high-risk condition.
